# Diagnostic imaging, therapeutic interventions and suggestions for thoracic duct congestion in postoperative hepatic lymphorrhea: a retrospective analysis of 20 cases

**DOI:** 10.1186/s12893-024-02650-6

**Published:** 2024-11-12

**Authors:** Xin Liu, Zhong Liu, Wenbin Shen, Song Xia, Yuguang Sun, Kun Chang, Jianfeng Xin, Ran An, Chen Liang, Chenxiao Zhou

**Affiliations:** 1 Department of Lymphatic Surgery, Affiliated Beijing Shijitan Hospital, Capital Medical University, Beijing, 100038 China; 2 Clinical Center for Lymphatic Disorders, Capital Medical University, Beijing, China

**Keywords:** Thoracic duct, Liver, Lymphorrhea, Lymphoscintigraphy, Lymphography

## Abstract

**Objective:**

To retrospectively evaluate thoracic duct (TD) congestion in hepatic lymphorrhea (HL) and propose treatment suggestions.

**Methods:**

Retrospectively analyze cases of postoperative HL admitted from August 2007 to November 2023. Twenty cases were enrolled and followed up. The medical history, ascites characteristics, lymphoscintigraphy, direct lymphangiography, and other clinical data were reviewed.

**Results:**

Twenty patients with ascites after cholecystectomy or radical gastrectomy were included. There were 15 patients with cirrhosis and 5 patients with hepatitis. Ascites were light yellow even if the patients had a non-low-fat diet. Triglyceride level mean of ascites was 0.61 ± 0.20 mmol/L. There were 94.1% (16/17) of patients whose ascitic cholesterol ≥ 45 mg/dL or SAAG < 11.0 g/L. Mild abdominal radioactivity was shown in 89.5% (17/19) patients. Left subclavian-jugular venous angle radioactivity was observed in 84.2% (16/19) patients. In 10% (2/20) cases, lipiodol presenting as oil droplets traveled upwards quickly and flowed into the vein rapidly. In 90% (18/20) cases, tortuous and dilated thoracic duct, stagnant lipiodol, and poor flow into the vein were demonstrated. One patient refused treatment and died soon. By thoracic duct outlet reconstruction combined with other treatments, 16 patients were cured and the ascites of another 3 patients were controlled.

**Conclusions:**

TD congestion and elevated lymphatic pressure could be caused by increased lymph flow and TD outlet stenosis. TD decompression by outlet reconstruction may be an alternative approach to HL.

**Supplementary Information:**

The online version contains supplementary material available at 10.1186/s12893-024-02650-6.

## Background

Hepatic lymphorrhea (HL) is a rare postoperative complication because of surgical injury to hepatic lymphatic ducts. It could occur after operations of the upper digestive system, such as cholecystectomy [1], radical gastrectomy [2], hepatectomy [3], and pancreatoduodenectomy [4, 5]. The patients presented with massive ascites. The main component of the ascites is hepatic lymph. By laboratory analysis, clear color, high concentration of protein, and constant low concentration of triglyceride (< 1.25 mmol/L) was found, which was different from chylous ascites. The preliminary clinical diagnosis of HL can be established by excluding all other potential causes of abdominal ascites. Percutaneous transhepatic lymphography can demonstrate hepatic lymphatic fistulae [5, 6]. The technique of interstitial embolization has been increasingly reported [3, 7–11]. But, there are no reliable data about the best way to treat HL.

We noticed thoracic duct congestion in HL during the diagnosis of postoperative lymphorrhea by lymphoscintigraphy and trans-pedal lymphangiography. Most of the patients with HL were reported with hepatitis or cirrhosis [7]. It is well known that lymph production and flow are greatly increased in patients with cirrhosis. Overproduction of hepatic lymph compromises lymphatic circulation. The thoracic duct (TD) receives lymphatic drainage from the liver. Stenosis of the TD lumen and the lymphovenous junction constrains the outflow of lymph. Impaired lymphatic circulation may contribute to postoperative lymphorrhea or chylorrhea.

Previously, cervical lymphovenous shunt was used for the treatment of ascites with cirrhosis [12], which resulted in clinical improvements in ascites and portal hypertension [13]. Recently, endovascular lymphatic decompression via thoracic duct stent placement for refractory ascites showed promising results in patients with contraindications for liver transplantation and transjugular intrahepatic portosystemic shunt (TIPS) [14]. We believe that thoracic duct decompression is beneficial to draining excessive lymphatic fluid from the liver and promoting closure of the leak site. We had explored thoracic duct outlet reconstruction for intractable ascites due to HL.

The objectives of this retrospective observational study were to evaluate radiological findings of lymphatic circulation, discuss the effect of thoracic duct congestion on HL, and describe our experiences of managing HL.

## Methods

### Patients

We retrospectively reviewed the medical records of adult patients with postoperative lymphorrhea admitted into the Department of Lymphatic Surgery, Capital Medical University Affiliated Beijing Shijitan Hospital, from August 2007 to November 2023. Patients were eligible for HL if matching the following criteria: 1) post-operative new-onset ascites after cholecystectomy (with or without exploration of the bile duct) or radical gastrectomy; 2) non-milky ascites, high protein level, and low triglyceride level (consistently < 1.25mmol/L) even if normal or high-fat diet; 3) excluding biliary fistula, pancreatic fistula, and malignant ascites. We also made the diagnosis if the leak site was confirmed in exploratory laparotomy because of resistance to medical treatments for cirrhotic ascites. The following exclusion criteria were used: 1) Cytopathology of ascites was not performed; 2) Lymphoscintigraphy, non-contrast MRL, or lymphangiography was not implemented. We collected data including demographics, clinical presentations, laboratory data, lymphatic imaging results, treatments, and outcomes.

### Laboratory analysis

Data of ascites including color, Rivalta test, differential cell count, chyle test, biochemical tests (adenosine deaminase, lactate dehydrogenase, total protein, albumin, cholesterol, and triglyceride), and cytopathology were collected. Biochemical data of peripheral blood were gathered. Serum-ascites albumin gradient (SAAG), Child–Pugh score, and MELD score were calculated.

### Lymphoscintigraphy, lymphangiography and CT, non-contrast magnetic resonance lymphangiography (MRL)

Patients underwent ^99m^Tc-DX lymphoscintigraphy imaging according to the protocol reported by Zhang et al. [15]. Whole-body images were acquired at 10 min, 1 h, 3 h, and 6 h after injection. The analysis of imaging characteristics focused on whether the lymphatic drainage was aberrant and whether there was abnormal radioactive accumulation in the abdominal cavity and abnormal radioactive tracer distribution in other parts. Abnormal radioactivity in the left subclavian-jugular venous angle suggests TD obstruction [16].

Trans-pedal lymphangiography with lipiodol (Guerbet, France) was done (referring to the review article by Pieper et al. [17]). We dynamically evaluated the flow of lymph by digital subtraction angiography (DSA). The flow rate of lipiodol and the morphology of the thoracic duct, especially the lymphovenous junction, were observed. The patients received CT scans before and after lymphangiography to detect the type and extent of lymphatic pathology. Non-contrast MRL of the patients were acquired. TD obstruction was defined as a visible narrowing and stasis of lipiodol around the ampulla [18].

### Treatment and follow-up

Conservative treatments, thoracic duct outlet reconstruction, or surgical ligation of the leak site with fibrin glue sprinkle via relaparotomy were considered as alternative options. Standardized conservative treatments covered avoidance or lessening of drainage, oral diuretics (furosemide and spironolactone), strict low-fat diet, and total parenteral nutritional support. If thoracic duct congestion or outlet obstruction was confirmed by lymphangiography, thoracic duct outlet reconstruction was performed. During the operation, the cervical thoracic duct and lymphatic trunks converging into the ampulla were exposed. Compression, angular malalignment, adhesion, and thicker fibrous tissue around the adventitia were removed. Improved lymphatic drainage into the vein was observed under the microscope. Follow-up of these patients was conducted via telephone.

### Statistics

Descriptive statistics (Mean ± standard deviation, number and proportions) were used. All statistical analyses were performed using IBM SPSS Statistics for Windows (version 22.0, IBM).

## Results

We included 20 patients with ascites following cholecystectomy (13 patients) or radical gastrectomy (7 patients). There were 15 patients with cirrhosis and 5 patients with hepatitis (1 patient with Niemann-Pick Disease, 2 patients with HBV hepatitis, 1 patient with alcohol hepatitis, and 1 patient with chemotherapeutic liver damage induced by Oxaliplatin, TS-1, and Nivolumab) Table 1.

**Table 1:** Patient characteristics

	Cholecystectomy	Radical gastrectomy	Total
Sex N (%)			
Male	6(46.2%)	6(85.7%)	12(60.0%)
Female	7(53.8%)	1(14.3%)	8(40.0%)
Age Mean (± SD)	49.3(3.1)	46.7(9.8)	48.4(10.5)
Child–Pugh N (%)			
A	1	3	4
B	12	4	16
Liver disease N (%)			
Alcohol	1(7.7%)	1(14.3%)	2(10.0%)
Virus	7(53.8%)	4(57.1%)	11(55.0%)
NASH	1(7.7%)	0	1(5.0%)
drug-induced liver injury	1(7.7%)	1(14.3%)	2(10.0%)
others	3(23.1%)	1(14.3%)	4(20.0%)
Hepatic cirrhosis N (%)			
Yes	12(92.3%)	3(42.9%)	15(75.0%)
No	1^a^(7.7%)	4(57.1%)	5(25.0%)
MELD Mean (± SD)	9.8(4.1)	9.2(4.5)	9.6(4.1)
Splenomegaly N (%)			
Yes	7(53.8%)	1(14.3%)	8(40.0%)
No	6(46.2%)	6(85.7%)	12(60.0%)
**Biology**			
Platelets Mean (± SD)	153(61.5)	280(91.5)	193(92.2)
PT in % Mean (± SD)	81.7(18.1)	83.0(17.1)	82.1(17.3)
Albumin in g/L Mean (± SD)	31.0(5.5)	31.5(8.4)	31.1(6.3)
Bilirubin in µmol/L Mean (± SD)	16.8(9.8)	18.0(15.4)	17.2(11.4)
Creatinin in µmol/L Mean (± SD)	84.7(37.5)	81.8(43.1)	83.8(38.1)
**Ascites**			
Triglyceride level in mmol/L Mean (± SD)	0.59(0.21)	0.66(0.16)	0.61(0.20)
Cholesterol level in mmol/L Mean (± SD)	1.40(0.79)	1.35(0.42)	1.39(0.67)
Albuminemia in g/L Mean (± SD)	20.9(5.3)	22.3(9.8)	21.4(6.9)
SAAG in g/L Mean (± SD)	9.7(4.6)	8.3(2.6)	9.3(4.1)

*SAAG* Serum-ascites albumin gradient of 17 patients

^a^Niemann-Pick Disease

The drains were kept open before referral to our department. The minimum and maximum drainage amounts were 800ml/d and 5000ml/d. The volume of drainage was greater in patients with advanced cirrhosis. The ascites appeared light yellow even if the patients had a non-low-fat diet. The mean triglyceride level in the ascites was 0.61 ± 0.20 mmol/L. The mean cholesterol level in the ascites was 1.39 ± 0.67mmol/L, and 60.0% (12/20) of patients ≥ 45 mg/dL. The mean albuminemia concentration in the ascites was 21.4 ± 6.9 g/L. The mean SAAG of 17 patients was 9.3 ± 4.1 g/L (3.7 ~ 17.6 g/L), and 76.5% (13/17) of patients < 11.0 g/L. There were 47.1% (8/17) of patients whose ascitic cholesterol ≥ 45 mg/dL and SAAG < 11.0 g/L. There were 94.1% (16/17) of patients whose ascitic cholesterol ≥ 45 mg/dL or SAAG < 11.0 g/L.

**Fig. 1:**
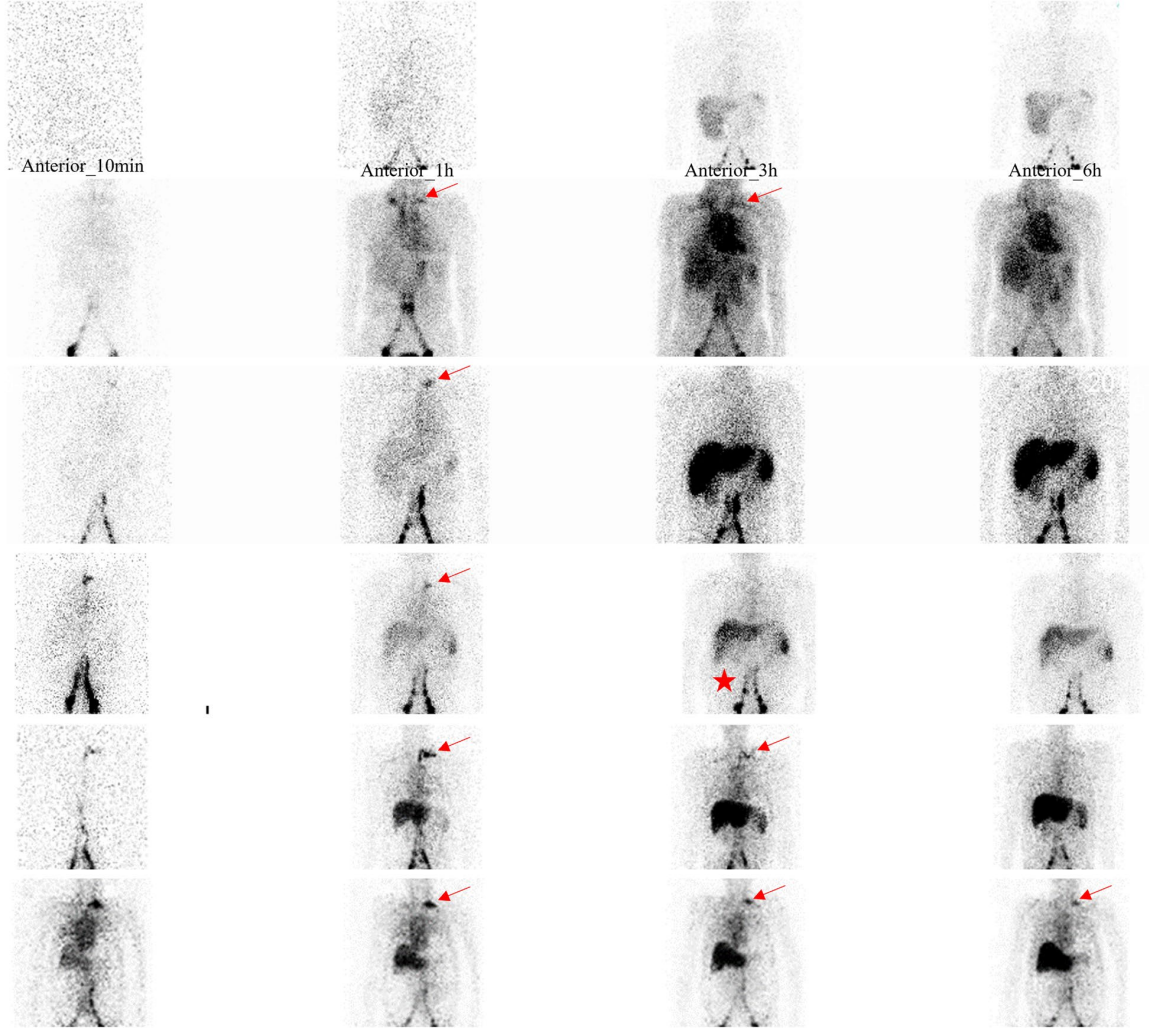
Features of Lymphoscintigraphy. Continuing or temporary left subclavian-jugular venous angle radioactivity (arrow). Mild abdominal radioactivity was shown (star)

Lymphoscintigraphy was performed on 19 patients. Mild abdominal radioactivity was shown in 89.5% (17/19) of the patients. The remaining 10.5% (2/19) did not show any abdominal radioactivity. Radioactivity in the left subclavian-jugular venous angle indicates thoracic duct congestion. Figure 1 illustrates the different types of lymph flow patterns in the thoracic duct. Continuing left subclavian-jugular venous angle radioactivity was observed in 63% (12/19) of the patients. Temporary left subclavian-jugular venous angle radioactivity was observed in 21% (4/19) patients. The remaining 16% (3/19) of the patients exhibited no radioactivity in the subclavian-jugular venous angle.

**Fig. 2:**
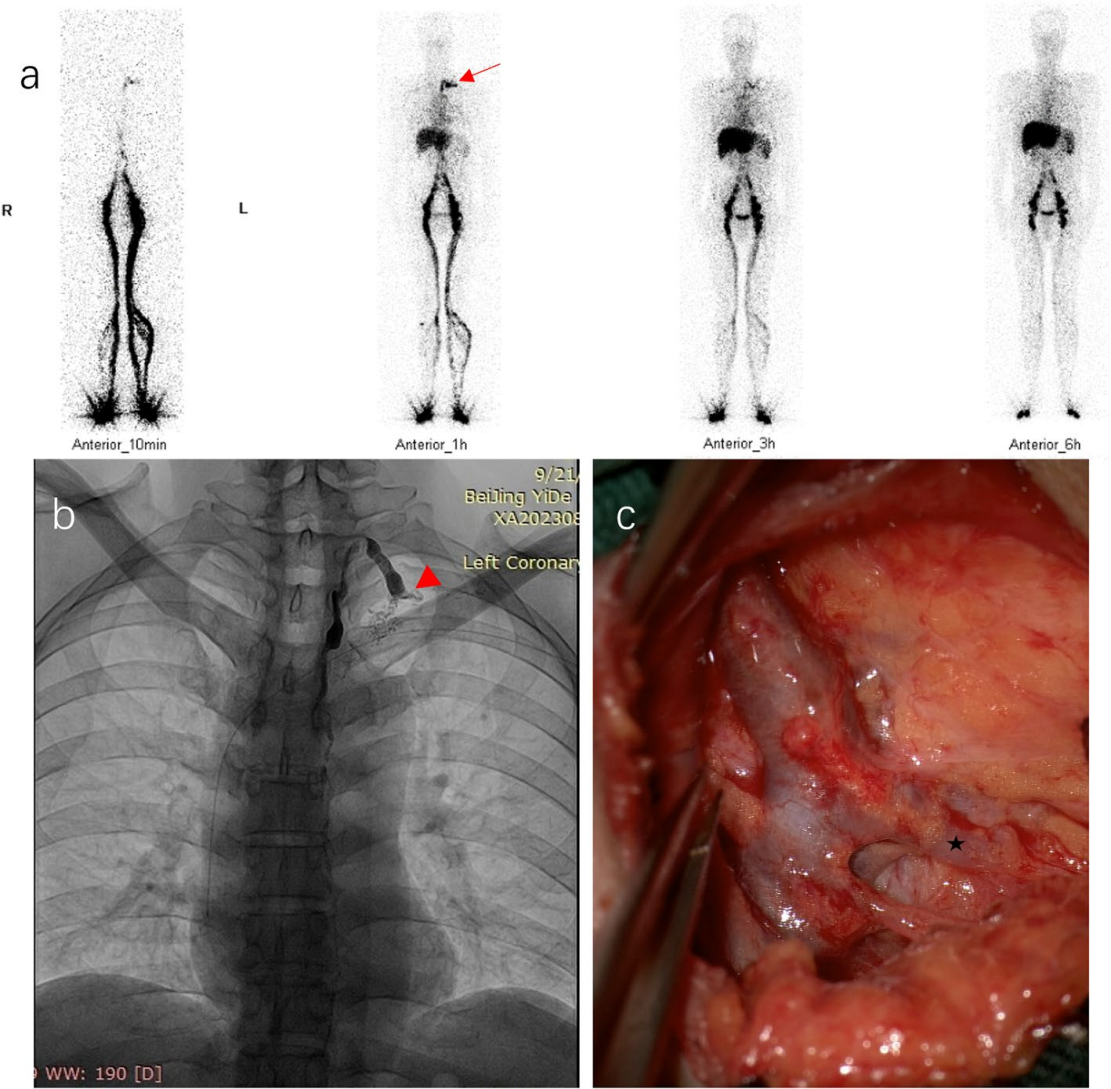
Functional obstruction of thoracic duct outlet. The patient suffered hepatic lymphorrhea after laparoscopic radical gastrectomy. Lymphoscintigraphy (**a**) showed temporary left subclavian-jugular venous angle radioactivity (arrow). Lymphangiography (**b**) demonstrated a distended cervical thoracic duct and ampulla (arrowhead), raised pressure in the duct, and increased flow rate into the vein. Lymph flow through thoracic outlets was mildly restricted and reflux was slight (arrowhead). The thoracic duct and subclavian lymphatic trunk (star) were distended (**c**)

**Fig. 3:**
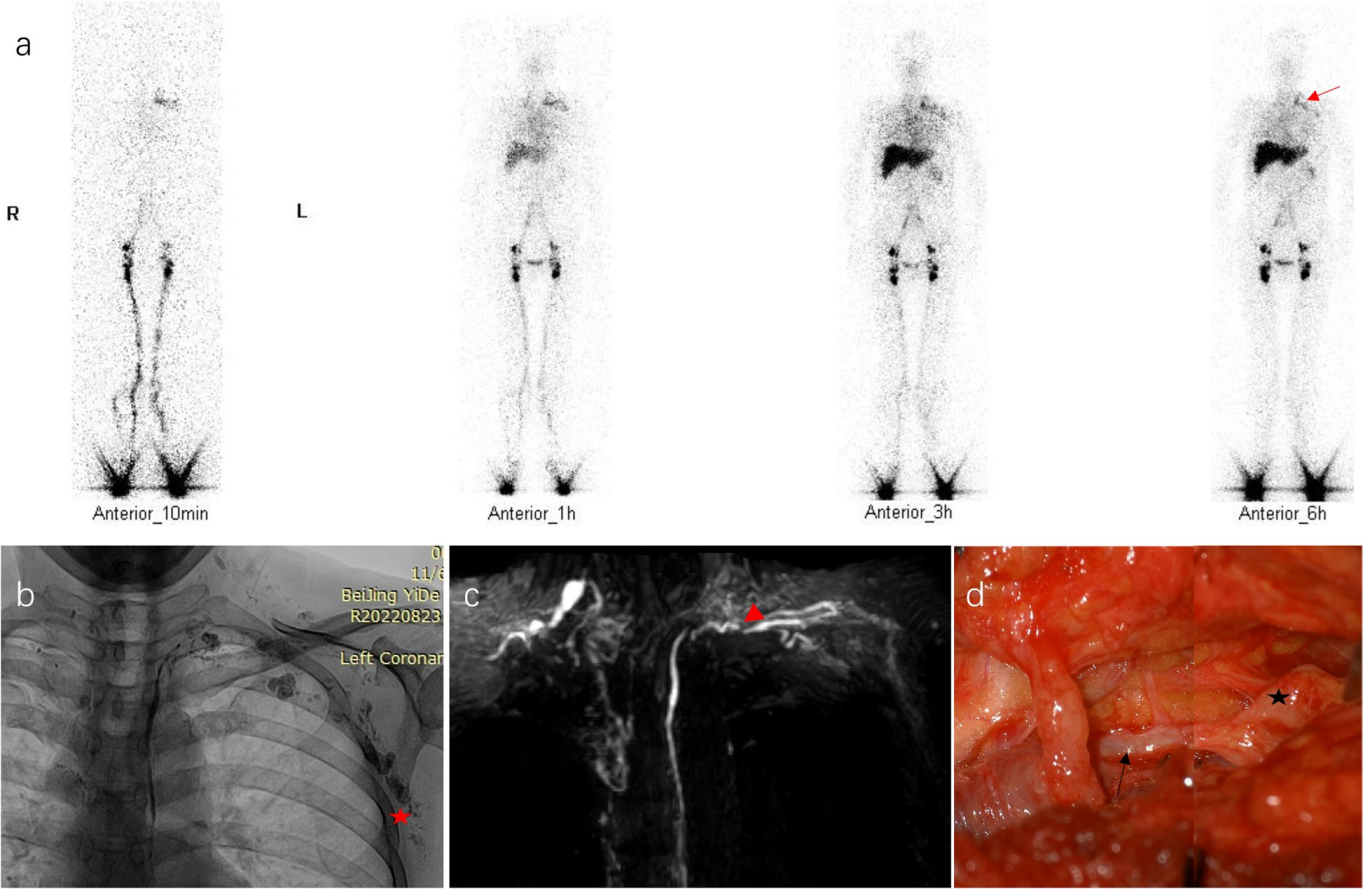
Anatomical obstruction of thoracic duct outlet. The patient with drug-induced liver injury suffered from hepatic lymphorrhea after laparoscopic cholecystectomy. Lymphoscintigraphy (**a**) showed continuing left subclavian-jugular venous angle radioactivity (red arrow). Lymphangiography (**b**) demonstrated massive reflux of lipiodol to the axilla (red star). The outlet was stenotic in anatomy and the ampulla was invisible (red arrowhead) (**c**). A branch of the thoracic duct (black star) converged into the subclavian lymphatic trunk and the confluence was segmentally stenotic (black arrow) (**d**)

All patients underwent trans-pedal lymphangiography. No contrast agent leaked into the abdominal cavity. In 10% (2/20) of cases, lipiodol appeared as oil droplets traveled upward quickly and flowed into the vein rapidly. In 90% (18/20) of cases, tortuous and dilated thoracic duct, stagnant lipiodol, and poor flow into the vein were demonstrated. In 5 of the 18 cases, no entry of the contrast medium into the vein was seen under DSA for 30 s. In 17 of the 18 cases, reflux of the contrast agent towards lymphatic vessels or trunks adjacent to the ampulla was noticed). Figures 2 and 3 respectively showed TD functional obstruction and anatomical obstruction (with TD outlet stenosis).

Nineteen cases received treatments, and none required paracentesis at the time of discharge. Sixteen patients were cured, whose ascites disappeared within 13 days to 6 months after discharge. Three patients had moderate ascites but did not need paracentesis after discharge.

In the group with ascites after cholecystectomy: 7 patients were cured after thoracic duct outlet reconstruction; 5 patients received combined thoracic duct outlet reconstruction and surgical ligation of leak site with fibrin glue sprinkle via relaparotomy, 3 of them were cured and 2 of them had controlled ascites; 1 patient was cured after combined thoracic duct outlet reconstruction and TIPS.

In the group with ascites after radical gastrectomy: 2 patients recovered after conservative treatments; 3 patients recovered after thoracic duct outlet reconstruction; 1 patient abandoned treatment and died soon.; 1 case received palliative peritoneovenous shunt, who suffered lower esophageal varices bleeding during hospitalization and had controlled ascites for 2 years.

## Discussion

In the pathophysiology of HL, thoracic duct congestion appears to play a role. The overproduction of hepatic lymph and the obstruction of thoracic duct outlets can lead to thoracic duct congestion and an elevated lymphatic venous pressure gradient (LVPG). Elevated LVPG may potentially be pathogenic [18].

Lymphatic imaging findings support thoracic duct congestion in HL. Left subclavian-jugular venous angle radioactivity was observed in 84% of patients. Thoracic duct outflow obstruction was evident in 90% of patients. There are two types of obstruction: i. Functional obstruction: The amount and rate of lymph flow increased tremendously, left subclavian-jugular venous angle radioactivity decreased over time, lymph flow through thoracic outlets was mildly restricted, and reflux was slight (see Fig. 2); ii. Anatomical obstruction: The amount and rate of lymph flow also increased, left subclavian-jugular venous angle radioactivity persisted in the 6-h imaging, lymph flow through thoracic outlets was severely limited, and reflux was significant, e.g., reflux to the axilla (see Fig. 3).

Cirrhosis and hepatitis are important clinical features of postoperative HL. As we know, the liver is an important organ producing lymph. The albumin-rich liver lymph flow multiplies even if a slight increase in the hepatic venous pressures [19]. In patients with cirrhosis, the volume of lymph flow in the thoracic duct is significantly increased [20]. Consequently, when the peri-portal lymphatic vessels, which drain 70–80% of hepatic lymph [21], are damaged, large volumes of lymph leak into the abdominal cavity. Due to high pressure and increased flow rate, it is refractory to make the leak site sealed by conservative treatments.

Despite this, appropriate conservative treatments (Table 2) are fundamental and effective, especially in patients without abdominal radioactivity. Firstly, minor negative fluid balance is preferred when total parenteral nutrition is utilized. Oral diuretic drugs, such as furosemide and spironolactone, can be beneficial. Second, negative pressure active suction should be avoided. Clamping the drainage tube as early as possible (early removal if no infection of the abdominal cavity), and abridging the frequency and the volume of drainage is recommended. Meanwhile, abdominal distension and circumference must be closely monitored. If prolonged conservative treatments proved ineffective, especially if the patient suffered from severe abdominal distension and required large and frequent drainage, surgical interventions should be considered.

**Table 2:** Treatment alternatives of hepatic lymphorrhea

Conservative treatments
Avoidance or abridgement of drainage or paracentesis
Oral diuretics such as furosemide and spironolactone
Strict low-fat diet
Total parenteral nutritional support with minor negative fluid balance
Non-conservative treatments
Thoracic duct outlet obstruction
Thoracic duct outlet reconstruction
relaparotomy or laparoscopy
Surgical ligation and fibrin glue sprinkle
Interventional Radiology
Transhepatic Lymphatic Embolization
Advanced Cirrhosis with portal hypertensive ascites
Transjugular intrahepatic portosystemic shunt
Peritoneovenous shunt

Thoracic duct decompression may contribute to alleviating HL with compromised outflow of lymph via the thoracic duct. The ascites may be resolved by treating the obstruction of TD, and surgical ligation of leakage may be avoided. In Fontan patients, elevated central venous pressure leads to lymphatic failure, and decompression of the thoracic duct outlet could relieve lymphatic failure symptoms [22, 23]. In patients with Klippel-Trenaunay Syndrome, abnormal activity in the left subclavian-jugular venous angle was observed in 53.6% (15/28) of patients, and thoracic duct decompression improved symptoms of lymphedema in the lower extremities [16]. Abhay Srinivasan et. al. reported alleviation of symptoms in five patients without active leak on imaging who underwent interventions solely to treat TD obstruction [18]. Additionally, we observed the disappearance of the ascites after thoracic duct decompression in eight patients with HL. Patients who underwent radical gastrectomy and chemotherapy are fragile. For these patients, thoracic duct outlet reconstruction is less invasive than surgical ligation.

Moreover, HL should be differentiated from portal hypertensive ascites. HL was first identified in patients with virus hepatitis B after gastrectomy for gastric cancer in Japan [7]. Most cases were secondary to radical D2 dissection in the hepatoduodenal ligament during procedures for gastric carcinoma [5, 7, 24]. The ascites resulted from surgical injury to the hepatic lymphatics. Ascitic cholesterol levels and SAAG can help detect non-portal hypertensive ascites [25]. Clear-colored ascites, ascitic cholesterol ≥ 45 mg/dL, SAAG < 11.0 g/L, a high concentration of protein, and a low concentration of triglyceride support the diagnosis of HL. The features of ascites from our cases were consistent with the literature.

The two disorders could synchronously occur in patients with portal hypertension. In advanced cirrhosis, the expansion of the lymphatic vasculature facilitates the flow of lymph back into the systemic circulation, which prevents fluid from accumulating. Cirrhotic ascites emerge when the compensatory lymphatic system is overworked [26]. Endovascular lymphatic decompression via TD stent placement was effective on refractory ascites in some patients with cirrhosis [14]. In this case series, two patients who underwent cholecystectomy had both portal hypertensive ascites and HL. The drainage was tremendous and their ascites resembled portal hypertensive ascites. After thoracic duct decompression, intractable ascites transformed into manageable ascites. The patients returned to normal life, even though the ascites persisted but required no further drainage.

This retrospective study is meaningful but has several limitations. Our hospital serves as a tertiary medical center for lymphatic disorders. Only intractable patients with HL are referred to our center. The analysis involved a small cohort of patients without a control group. This may limit the generalizability of our findings to all patients with HL. Additionally, transjugular lymphangiography, transhepatic lymphangiography and interstitial embolization, and laparoscopic ligation of the leak site with fibrin glue sprinkle were not utilized in our clinical practices. The evidence is absent that thoracic duct congestion was improved and outlet obstruction was eliminated. Furthermore, the small sample size limits the applicability of statistical tests. Prospective and multicentric randomized clinical trials should be designed.

## Conclusion

TD congestion may have a potential role in the pathophysiology of HL. TD congestion and elevated lymphatic pressure could be caused by increased lymph flow and TD outlet stenosis. TD outflow obstruction can be classified into functional obstruction and anatomical obstruction, which includes extrinsic compression or idiopathic TD outlet stenosis. TD decompression by outlet reconstruction may serve as an alternative approach to HL.

## Supplementary Information

Supplementary Material 1.

## Data Availability

The datasets used and/or analyzed during the current study are available from the corresponding author on reasonable request.

## Abbreviations

HL: Hepatic Lymphorrhea
TD: Thoracic Duct
TIPS: Transjugular Intrahepatic Portosystemic Shunt
SAAG: Serum-Ascites Albumin Gradient
MRL: Magnetic Resonance Lymphangiography
LVPG: Lymphatic Venous Pressure Gradient
DSA: Digital Subtraction Angiography
