# Supplementary material for: Diagnostic imaging, therapeutic interventions and suggestions for thoracic duct congestion in postoperative hepatic lymphorrhea: a retrospective analysis of 20 cases
Source: BMC Surg. 2024 Nov 12;24:352. doi: 10.1186/s12893-024-02650-6 (PMC11556192; doi:10.1186/s12893-024-02650-6)

## Slide 1
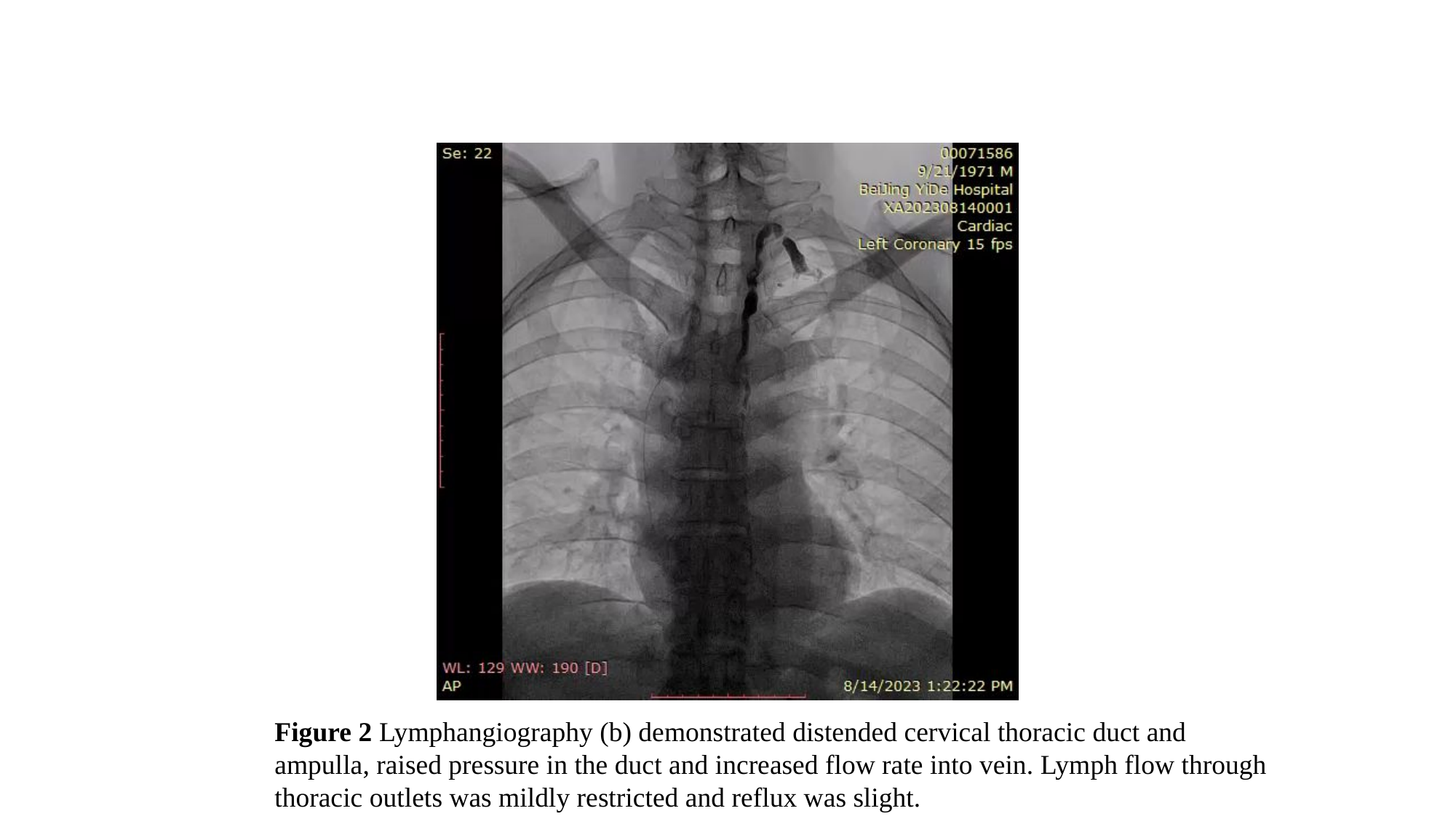

Figure 2 Lymphangiography (b) demonstrated distended cervical thoracic duct and ampulla, raised pressure in the duct and increased flow rate into vein. Lymph flow through thoracic outlets was mildly restricted and reflux was slight.

## Slide 2
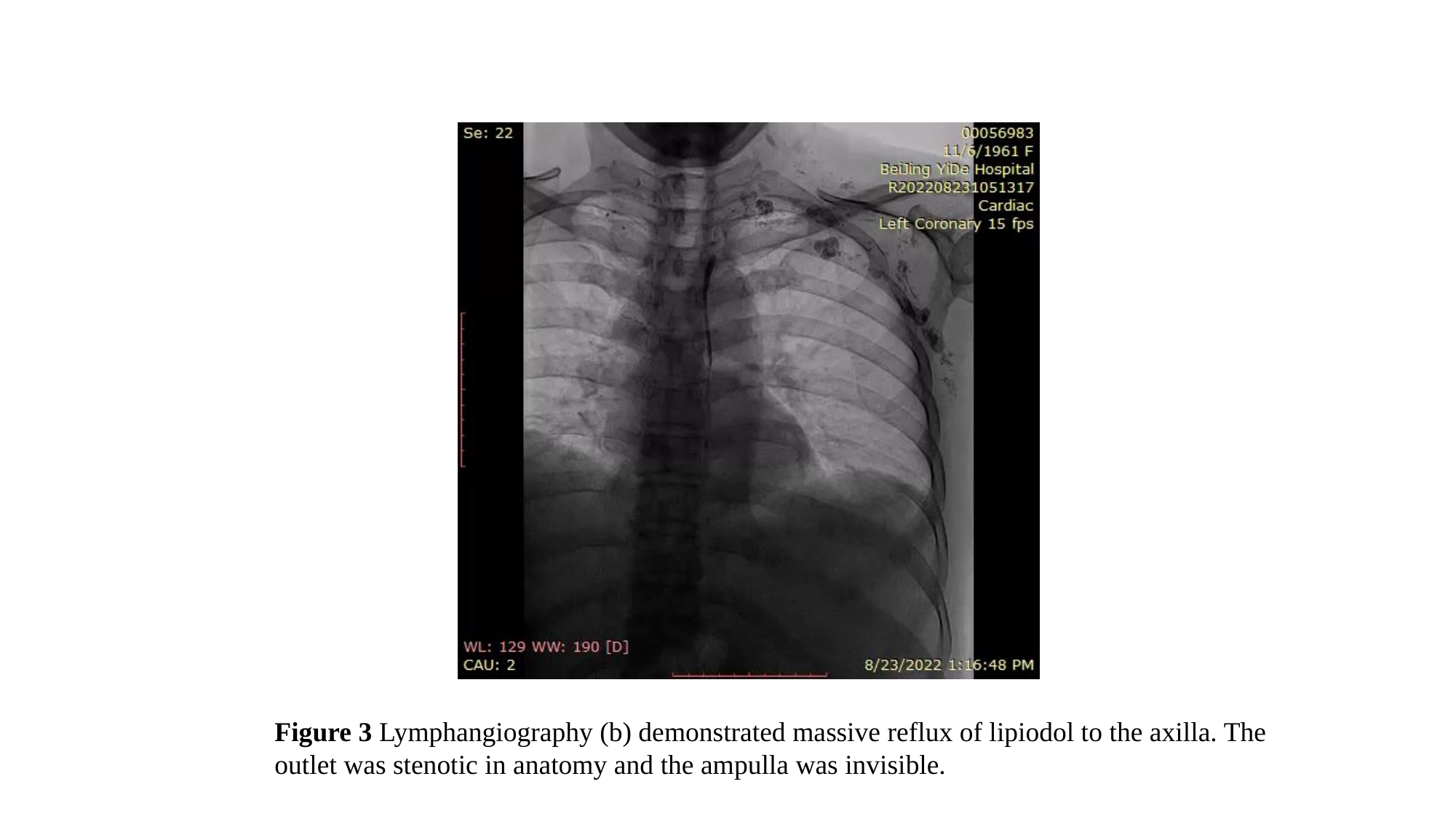

Figure 3 Lymphangiography (b) demonstrated massive reflux of lipiodol to the axilla. The outlet was stenotic in anatomy and the ampulla was invisible.

## Slide 3
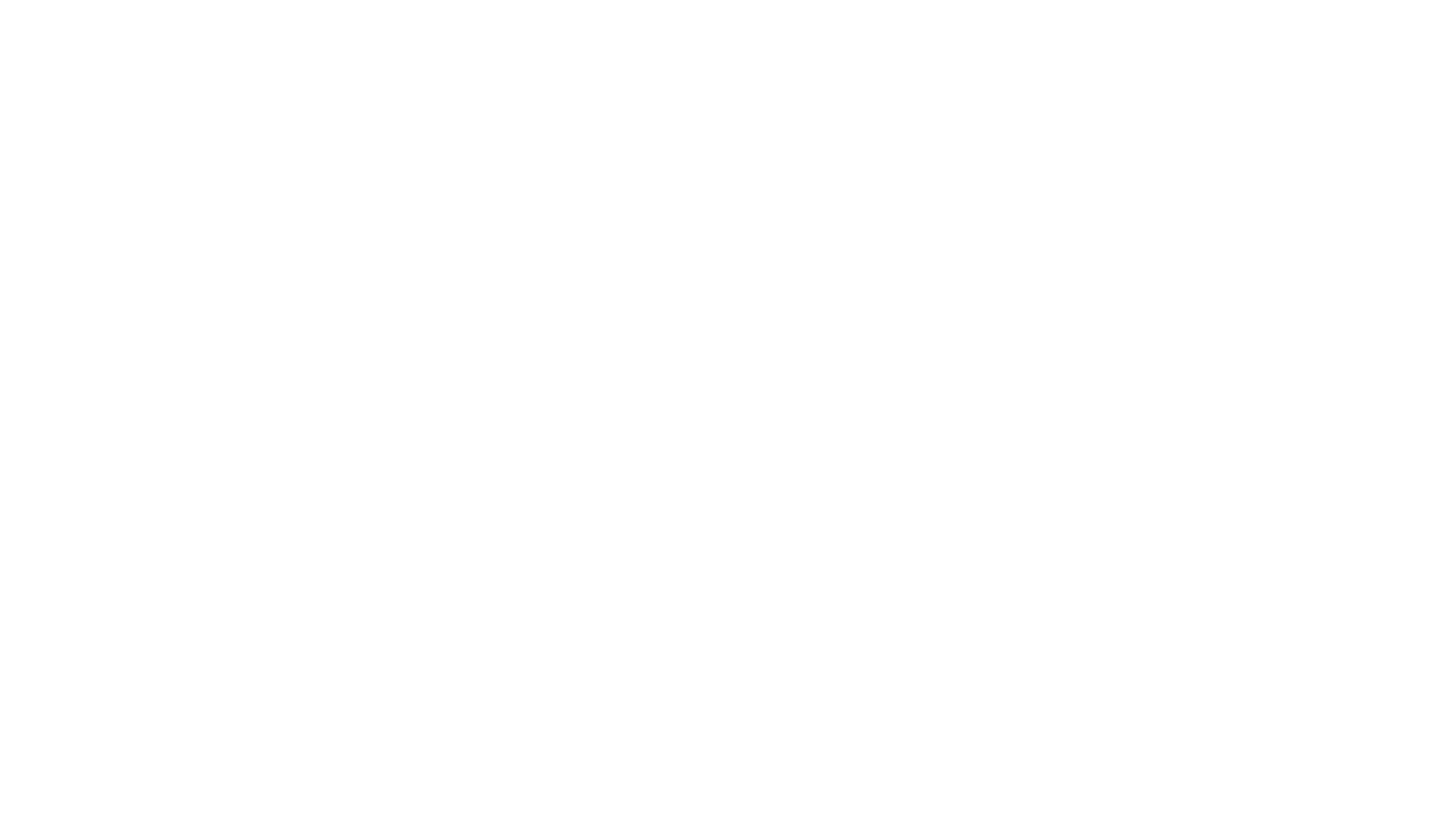

Supplement: Supplementary file 1 — Supplementary Material 1. [file 12893_2024_2650_MOESM1_ESM.pptx]
